# Preventive Roles of Rice-*koji* Extracts and Ergothioneine on Anxiety- and Pain-like Responses under Psychophysical Stress Conditions in Male Mice

**DOI:** 10.3390/nu15183989

**Published:** 2023-09-14

**Authors:** Kajita Piriyaprasath, Yoshito Kakihara, Atsushi Kurahashi, Mayumi Taiyoji, Kazuya Kodaira, Kotaro Aihara, Mana Hasegawa, Kensuke Yamamura, Keiichiro Okamoto

**Affiliations:** 1 Division of Oral Physiology, Faculty of Dentistry, Niigata University Graduate School of Medical and Dental Sciences, Niigata 951-8514, Japan or kajitap@nu.ac.th (K.P.); mana@dent.niigata-u.ac.jp (M.H.); yamamurak@dent.niigata-u.ac.jp (K.Y.); 2 Department of Restorative Dentistry, Faculty of Dentistry, Naresuan University, Phitsanulok 650000, Thailand; 3 Division of Dental Pharmacology, Faculty of Dentistry, Niigata University Graduate School of Medical and Dental Sciences, Niigata 951-8514, Japan; kakihara@dent.niigata-u.ac.jp; 4 Sakeology Center, Niigata University, Niigata 951-8514, Japan; 5 Hakkaisan Brewery Co., Ltd., Minamiuonuma, Niigata 949-7112, Japan; a.kurahashi@hakkaisan.jp (A.K.); k.kodaira@hakkaisan.jp (K.K.); 6 Food Research Center, Niigata Agricultural Research Institute, Kamo 959-1381, Japan; taiyoji.mayumi@pref.niigata.lg.jp (M.T.); aihara.kotaro@pref.niigata.lg.jp (K.A.); 7 Division of General Dentistry and Dental Clinical Education Unit, Faculty of Dentistry, Niigata University Graduate School of Medical and Dental Sciences, Niigata 951-8514, Japan

**Keywords:** Rice-*koji*, ergothioneine, anxiety, pain, SH-SY5Y cell

## Abstract

This study determined the effect of daily administration of Rice-*koji* on anxiety and nociception in mice subjected to repeated forced swim stress (FST). In a parallel experiment, it was determined whether ergothioneine (EGT) contained in Rice-*koji* displayed similar effects. Anxiety and nociception were assessed behaviorally using multiple procedures. c-Fos and FosB immunoreactivities were quantified to assess the effect of both treatments on neural responses in the paraventricular nucleus of the hypothalamus (PVN), nucleus raphe magnus (NRM), and lumbar spinal dorsal horn (DH). FST increased anxiety- and pain-like behaviors in the hindpaw. Rice-*koji* or EGT significantly prevented these behaviors after FST. In the absence of formalin, both treatments prevented decreased FosB expressions in the PVN after FST, while no effect was seen in the NRM and DH. In the presence of formalin, both treatments prevented changes in c-Fos and FosB expressions in all areas in FST mice. Further, in vitro experiments using SH-SY5Y cells were conducted. Rice-*koji* and EGT did not affect cell viability but changed the level of brain-derived neurotrophic factor. In conclusion, Rice-*koji* could reduce anxiety and pain associated with psychophysical stress, possibly mediated by the modulatory effects of EGT on neural functions in the brain.

## 1. Introduction

The management of mental illness and chronic pain induced by psychological stress could rely on the use of drugs. However, the effectiveness of drug treatments is inconsistent. Thus, better ways to cope with psychological stress daily are important for preventing anxiety and chronic pain because psychological stress is less avoidable in our life. Lifestyle medical approaches such as nutrition inventions have been shown to control psychological stress-related disorders [1].

Lately, consumers have been preferring natural foods, including rice-fermented foods, because of the increased interest in safer diets in Japan [2,3]. Rice-*koji*, made from *Aspergillus oryzae* (yellow *koji* mold), is a readily accessible contributor to nutrition and has been used as a source of rice-fermented food [4,5,6]. The beneficial effects of Rice-*koji* are due to the abundance of substances contained in it that have the potential to regulate bodily function [7]. Our previous studies indicated that rice-fermented beverages and foods such as sake and sake lees, which are made from *Aspergillus oryzae*, could prevent psychophysical distress and nociception [8,9]. Sake lees is a byproduct generated in the production process of Japanese sake, while Rice-*koji* is a starter in the production of sake [10]; however, the beneficial effects of Rice-*koji* on psychological stress are unclear. 

Several bioactive compounds contained in Rice-*koji* can regulate body functions [11]. For example, ergothioneine (EGT), contained in Rice-*koji*, is a derivative of an amino acid that is considered to provide health benefits [12]. Fermented rice with *Aspergillus oryzae* is an EGT-rich food [13], while EGT has the potential to prevent anxiety- [14] and pain-like [15,16] behaviors due to influences on neural responses in the brain [14].

The first aim of this study was to determine whether Rice-*koji* extracts (Rice-*koji*) can prevent anxiety- and pain-like behaviors under psychophysical stress conditions. In a parallel study, the effects of EGT on these responses were evaluated. These experiments could elucidate the possible molecular basis for the modulatory effects of Rice-*koji* on psychophysical stress responses in part. A paradigm of repeated forced swim stress (FST) was conducted to develop a psychophysical stress model in mice [17]. Multiple behavioral procedures were employed to evaluate the status of psychological stress, allowing us to assess the different aspects of psychological domains [18]. Further, the effects of Rice-*koji* and EGT on cognitive functions were also determined because psychological stress can cause cognitive impairments [19]. The novel object recognition (OR) test was conducted to assess short-term memory and learning [20,21]. Further, the effect of Rice-*koji* and EGT on pain-like behaviors under FST conditions was determined. A report revealed the inhibitory roles of EGT on neuropathic pain-like behaviors [15]; however, it remains unknown whether Rice-*koji* and EGT have inhibitory effects on nociception associated with psychological stress conditions. 

The second aim of this study was to determine the effect of Rice-*koji* and EGT on neural responses indicated by c-Fos and FosB immunoreactivities, markers for neural activity, in the brain. This study focused on several areas, including the paraventricular nucleus in the hypothalamus (PVN), nucleus raphe magnus (NRM), and lumbar spinal dorsal horn (DH) [22]. The PVN is one of the critical regions for regulating stress reactions [23,24], and dysfunction of the PVN can mediate anxiety-like behaviors. The NRM and DH are well documented as regulating nociceptive processing [9,25,26], and improvements in neural functions in the NRM and DH can decrease stress-induced nociception [8,27,28]. Notably, the NRM maintains neuronal connections with most pain-engaged areas in the brain [25]. The NRM receives neural inputs from the PVN and provides the descending inputs to the DH [29,30]. Previously, we reported that sake lees could regulate neural functions in the NRM and DH under stress conditions [9]. However, it is unknown whether this is the case for Rice-*koji* and EGT. 

This study also determined the potential effects of Rice-*koji* and EGT on neural responses using human neuroblastoma cells. SH-SY5Y cells, so-called human neuron-like cells, are used as an in vitro model for neural disorders [31,32,33,34]. First, the cell viability of SH-SY5Y cells was assessed using an MTT assay, which allowed us to evaluate whether Rice-*koji* itself has any negative effects on cell survival. Next, the level of brain-derived neurotrophic factor (BDNF) of the SH-SY5Y cells was assessed in the presence of Rice-*koji* or EGT. Because changes in BDNF signaling can cause psychological illness and chronic pain [35], measurements of the level of BDNF in SH-SY5Y cells supported the idea that Rice-*koji* and EGT exert modulatory effects on neural functions at the cellular level.

These notions indicate that Rice-*koji* could prevent anxiety and nociceptive responses associated with psychophysical stress through the modulation of neural responses in the brain due to the actions of EGT.

## 2. Materials and Methods

### 2.1. Preparation of Rice-koji Extracts (Rice-koji) and Ergothioneine (EGT)

Rice-*koji* was provided by the local sake brewery (HAKKAISAN BREWERY Co., Ltd., Niigata, Japan) and dissolved in PBS buffer at 0.5 g/mL concentration. Then, the supernatants after centrifugation (5000 rpm, 30 min) were filtered through a microfilter with a pore size of 0.22 µm and were used as a high dose of Rice-*koji* extracts (Rice-*koji*). The high dose of Rice-*koji* (High dose) was further diluted with PBS ×0.1, which was used as a low dose of Rice-*koji* (0.05 g/mL, Low dose). Rice-*koji* was stored at −20 °C until use. EGT (Nagara Science Co., Ltd., Nagoya, Japan) was diluted in saline, and 0.1 mg/kg and 1 mg/kg EGT were employed [15].

### 2.2. Quantification of EGT in Rice-koji

Rice-*koji* (High dose: 0.5 g/mL) was diluted with 50% acetonitrile solution and filtered through a 0.2 μm membrane filter. The quantitative value of the sample was obtained by the internal standard method. EGT was quantified by liquid chromatography–tandem mass spectrometry (LC-MS/MS). Prominence UFLC (Shimadzu Corporation, Kyoto, Japan) was used as the liquid chromatography instrument, and a ZORBAX Rx-SIL (150 mm × 2.1 mm, 5.0 µm particle size) column was used at 40 °C. The injection volume was 5 µL. Mass spectrometry was performed using a 4500 Qtrap (AB SCIEX, Tokyo, Japan) with electrospray ionization.

### 2.3. In Vivo Study

#### 2.3.1. Animals

We employed 200 male C57BL/6J mice. All animal experimental protocols were reviewed and approved by the Intramural Animal Care and Veterinary Science Committee of Niigata University (permit number: SA00611) and performed in accordance with the Guiding Principles for Care and Use of Laboratory Animals (National Institutes of Health). Male mice (C57BL/6J, Charles River Laboratories, 20–25 g) were employed. Mice were kept in cages (4 mice per cage) made of transparent acrylic (30 × 20 × 15 cm) with free access to food and water. Cages were kept in a temperature- and humidity-controlled environment with a 12:12 light/dark cycle (light phase: 7:00–19:00).

#### 2.3.2. Psychophysical Stress Model

**Figure 1:**
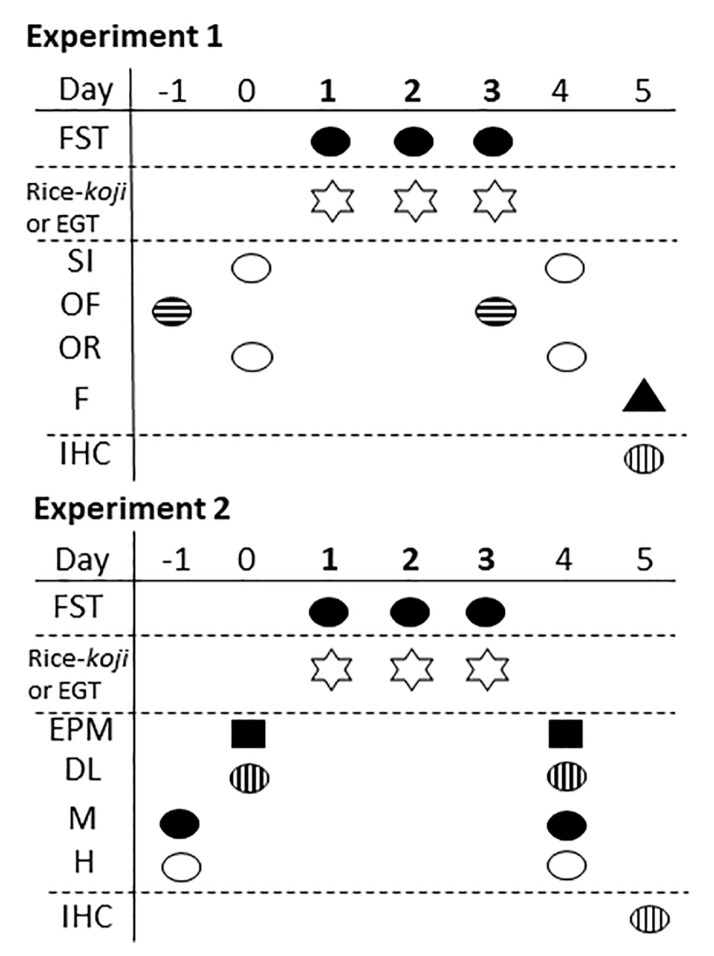
All mice (*n* = 200) were distributed into either Experiment 1 (*n* = 94) or 2 (*n* = 106). In both Experiments 1 and 2, mice were subjected to Sham (*n* = 100) or forced swim stress treatment (FST, *n* = 100) on Days 1, 2, and 3 (5 min/day). Either saline, Rice-*koji* extracts (Rice-*koji*), or ergothioneine (EGT) was administered orally 30 min and 6 h after daily conditionings of Sham or forced swim stress. In Experiment 1, the open field (OF) test was conducted on Day −1 and Day 3, while the social interaction (SI) and novel object recognition (OR) tests were conducted on Day 0 and Day 4. On Day 5, nociceptive responses were quantified by the hindpaw formalin behavioral tests and c-Fos and FosB immunohistochemistry (IHC). In Experiment 2, the elevated plus maze (EPM) and dark and light box (DL) tests were conducted on Day 0 and Day 4. The mechanical and thermal sensitivities were assessed in the hindpaw on Days −1 and 4. The IHC for c-Fos and FosB expression was conducted on Day 5.

This study employed a modified psychophysical model of rodents, which displayed a sustained behavior of despair for 1–3 weeks [17]. A repeated forced swim stress (FST) paradigm was employed to induce psychophysical stress conditions. Mice were placed and forced to swim in a plastic cylinder (diameter 15 cm, height 40 cm) containing water (25–30 °C) for 5 min/day between 8.30 a.m. and 9.30 a.m. for 3 days (Days 1, 2, and 3, Figure 1). After each FST, mice were dried in a warm environment. Sham mice were placed in the empty cylinder (diameter 15 cm, height 20 cm) filled with no water for 5 min. Sham and FST mice were housed in different cages.

#### 2.3.3. Oral Administration of Rice-*koji* and EGT

Rice-*koji* (Low or High doses) and EGT (0.1 or 1.0 mg/kg) were administered orally at 0.2 mL/mouse through a stomach tube 30 min and 6 h after each Sham or FST experiment on Days 1, 2, and 3 (Figure 1).

#### 2.3.4. Experimental Design

Mice were separated into two experiments: Experiment 1 and Experiment 2 (Figure 1). In Experiment 1 (*n* = 94: Sham, *n* = 45, FST, *n* = 49), the social interaction (SI), open field (OF), novel object recognition (OR), and hindpaw formalin tests were conducted. In Experiment 2 (*n* = 106: Sham, *n* = 55, FST, *n* = 51), the elevated plus maze, dark and light box, and pain-like behavioral tests evoked by heat and mechanical pressure stimulation were conducted. In each experiment, Sham and FST mice were divided into 5 groups as follows: vehicle (saline)-, Rice-*koji* (Low and High dose)-, and EGT (0.1 and 1.0 mg/kg)-treated groups. For the immunohistochemical (IHC) experiments, several mice were randomly selected and divided into two groups with or without formalin injection into the left hindpaw on Day 5. The experimenters who conducted the following behaviors and IHC experiments were blind to treatments. The number of animals employed in each experiment is present in the figure legends.

#### 2.3.5. Anxiety and Cognitive Assessments

Multiple behavioral procedures were employed to assess the effects of Rice-*koji* and EGT on the conditions of psychological distress and recognition (Figure 1).

1. Immobility time during FST

During the 5 min FST, non-swimming time (immobility time) was recorded. Immobility behaviors were when the mouse remained afloat in the water without struggling and only made the movements required to keep its head just above the water. The experimenter was blinded to all treatments.

2. Open field (OF) test

The OF test was employed to measure locomotor activity on Day −1 and Day 3. Mice were placed in a clear acrylic cage with black frosting Plexiglas floor (45 × 45 × 30 cm) for 5 min. The total movement distances and the time spent in the center zone area were measured using digital counters with infrared sensors (SCANET MV-40 MOV, MELQUEST Co., Ltd., Toyama, Japan). On Day 3, the OF test was conducted 5–6 h after the FST conditioning. The experimenter was blinded to all treatments.

3. Social interaction (SI) test

The SI was conducted on Day 0 and Day 4. Mice were put in the center of a clear acrylic cage with a black frosting Plexiglas floor that served as an open field area (45 × 45 × 30 cm). The wide area was divided into two interest zones: the interaction zone (IZ) and corner zone (CZ). The SI test consisted of two sessions (2.5 min each). For the first session, mice were placed in the open field without the male stranger mouse (Institute of Cancer Research (ICR) mouse, 30–35 g) in the IZ. Next, 1 min later, the ICR mouse was placed in the middle of IZ inside the perforated acrylic enclosure. In both sessions, the time spent in the IZ was recorded using digital counters with infrared sensors (SCANET MV-40 MOV, MELQUEST Co., Ltd., Toyama, Japan). The experimenter was blinded to all treatments.

4. Elevated plus maze (EPM) test

On Day 0 and Day 4, a mouse was placed in the center of a maze that consisted of 2 opened and 2 closed arms. The maze was raised around 50 cm above the ground (PM-DR25M-S, BrainScience Idea. Co., Ltd., Osaka, Japan). The duration of time spent in open arms was recorded for 5 min. The experimenter was blinded to all treatments.

5. Dark and light box (DL) test

On Day 0 and Day 4, a mouse was placed in a dark and light box with two compartments, one a light chamber and the other a dark chamber, which were joined by an opening. Mice were initially placed in the light chamber, and the behavior of the mouse was observed for 15 min. The time spent in each chamber was measured by digital counters with infrared sensors (SCANET MV-40 MOV, MELQUEST Co., Ltd., Toyama, Japan). The DL test was conducted 2 h after the EPM tests. The experimenter was blinded to all treatments.

6. Novel object recognition (OR) test

OR is a measure of recognition memory and primarily relies on the natural tendency of mice to spend more time exploring a novel object than a previously encountered one [20,21]. The OR test had two trials. In the first trial, mice were placed in an open field area and allowed to discover two similar objects (familiar objects), set in the A and A′ areas, for 5 min before being returned to their cage. Sixty minutes later, mice were brought to the testing area again and exposed to a familiar (in the A area) and novel object (in the B area) in the second trial. On Day 0 and Day 4, the time spent in each area was measured in both trails (5 min each) using digital counters with infrared sensors (SCANET MV-40 MOV, MELQUEST Co., Ltd., Japan). The experimenter was blinded to all treatments.

#### 2.3.6. Assessment of the Hindpaw Nociception (Pain-like Behaviors)

Effects of Rice-*koji* and EGT on the pain-like behaviors evoked by three different modalities, heat, mechanical pressure, and formalin, were measured in Sham and FST mice

1. Hindpaw heat sensitivity

Heat withdrawal latency was measured on Day −1 and Day 4 (plantar test, Hargreaves method [36], Ugo Basile, Italy). A mouse was placed in a transparent plexiglass chamber and acclimated for > 30 min. An infrared ray beam was emitted from under the glass floor and focused on the middle part of the plantar surface of the left hindpaw. The hindpaw withdrawal latency was measured. Heat stimuli were given twice to each mouse at intervals of 5 min. The average of two tests was recorded as the heat withdrawal latency. The experimenter was blinded to all treatments.

2. Hindpaw mechanical pressure sensitivity

The Randall–Selitto (Analgesy-Meter, Ugo Basile, Italy [37]) paw-pressure test was employed to assess nociceptive responses of the left hindpaw in mice on Day −1 and Day 4. This device applies linearly increased force (in grams) until the mouse produces a response characterized by hindpaw retraction or vocalization as mechanical hypernociception. This test was conducted one hour after the heat stimulation. The pressure stimuli were given twice to each mouse at intervals of 5 min. The average data of the two tests were recorded as the pressure threshold. The experimenter was blinded to all treatments.

3. Hindpaw formalin test

The hindpaw formalin test was conducted on Day 5 to assess the nocifensive behaviors of mice. Two microliters of 2.5% formalin was injected subcutaneously into the left hindpaw, and then mice were returned to the cage for a 45 min observation period. A stopwatch was used for 15 consecutive 3 min intervals to record the time spent on licking behaviors at the injected hindpaw. Licking behavior responses were divided into 2 phases—early phase (0–9 min) and late phase (9–45 min)—to analyze the results. The experimenter was blinded to all treatments.

#### 2.3.7. c-Fos and FosB Immunohistochemistry (IHC)

On Day 5, 5–10 animals were randomly selected in each treatment group and subjected to c-Fos and FosB immunohistochemical experiments 24 h after the last behavioral tests conducted on Day 4 (Figure 1). Animals employed in Experiment 1 were sacrificed 1.5 h after the hindpaw injection of 2.5% formalin (2 µL). All mice were deeply anesthetized with three mix agents prepared with 0.3 mg/kg of medetomidine (Domitor, Nippon Zenyaku Kogyo, Fukushima, Japan), 4.0 mg/kg of midazolam (Midazolam, SANDOZ, Princeton, USA), and 5.0 mg/kg of butorphanol (Vetorphale, Meiji Seika Pharma, Tokyo, Japan) and perfused transcardially with 20 mL heparinized saline, followed by 4% paraformaldehyde in 0.1 M phosphate-buffered saline (PBS) at pH 7.4. The brain and lumbar spinal cord were removed and postfixed in 4% paraformaldehyde at 4 °C for a few days and stored in 30% sucrose in 0.1 M PBS (pH 7.4) overnight. The spinal cord (L4), caudal brainstem, including the nucleus raphe magnus, and brain, including the hypothalamus, were cut into transverse sections (thickness 40 µm) using a microtome (REM-710, RETRATOME, YAMATO, Saitama, Japan). Sections were collected in five wells containing cold 0.1 M PBS and were used for c-Fos and FosB immunoreactivities. After sections were rinsed with PBS several times, they were incubated in affinity-purified rabbit c-Fos monoclonal antibody (9F6, 1:1000, Cell Signaling Technology, Danvers, MA, USA) or FosB (1:2000, Abcam, Cambridge, UK) in PBS with Tween 20 (PBST) and 5% normal goat serum overnight at room temperature. Sections were then washed with PBS twice, and then added to biotinylated goat anti-rabbit IgG antibody (1:200, Vector Laboratories, Burlingame, CA, USA) and 5% NGS in PBST for 2 h at room temperature. After washing sections with PBS twice, an avidin–biotin–peroxidase complex (Vector Laboratories, USA) was added to PBS for 1 h. Sections were washed with Tris-buffered saline (TBS) twice and then incubated in diaminobenzidine and nickel solution activated by 0.01% peroxidase. After visualizing c-Fos or FosB immunoreactivity, all sections were washed with TBS, mounted on the glass slides, and dried. Then, sections on the glass slides were dehydrated in the ethanol, cleared in xylene, and then cover slipped. The c-Fos- and FosB-positive cells in the paraventricular nucleus of the hypothalamus (PVN) and nucleus raphe magnus (NRM) were quantified in sections, having as reference the following anterior–posterior coordinates [38]: bregmas: PVN (−1.92 mm), NRM (−6 to −6.2 mm, [39]). The average number of positive cells in the PVN, NRM, and DH are presented. Cell counts were conducted manually under bright-filed illumination, and c-Fos- and FosB-positive cells were distinguished as homogenous black–gray elements with well-defined borders. Specific immunoreactivity was absent after the omission of the primary antibody. The cell count was conducted bilaterally in the PVN, and the average number of positive cells obtained from both sides is presented. In the L4, sections from laminae I–II and III–V ipsilateral to the hindpaw stimulation or on the left side in the absence of hindpaw stimulation were analyzed separately. Three sections were randomly selected from each area, and the number of c-Fos- and FosB-positive cells was counted. The quantitative analysis was conducted by an examiner who was blinded to treatment allocation.

### 2.4. In Vitro Study

#### 2.4.1. Cell Culture of SH-SY5Y

SH-SY5Y cells are often employed as the in vitro models for anxiety [40] and pain [33,41]. The SH-SY5Y cell lines (ATCC, Manassas, VA, USA) were maintained in DMEM/Ham’s F12 (FUJIFILM Wako Pure Chemical Corporation, Osaka, Japan) supplemented with 2.5 mM L-glutamine, 10% fetal bovine serum, and antibiotics—100 U/mL of penicillin and 100 μg/mL of streptomycin (FUJIFILM Wako Pure Chemical Corporation, Osaka, Japan)—in a humidified atmosphere of 5% CO_2_ at 37 °C. For the neuronal induction of SH-SY5Y cell lines, the culture medium was replaced with a differentiation medium containing 10 μM of all-trans retinoic acid (FUJIFILM Wako Pure Chemical Corporation, Osaka, Japan) in the presence of Rice-*koji* extracts (10 or 500 µg in 1 mL PBS), EGT (10 or 100 µg in 1 mL PBS), or PBS alone. The differentiation medium was replaced every 5 days over 4 weeks of incubation [42].

#### 2.4.2. Assessment of Cell Viability

An MTT assay was conducted. MTT solution was added at a final concentration of 500 μg/mL to the SH-SY5Y cells cultured in 6-well plates 28 days after the induction of differentiation and incubated for 3 h in a humidified atmosphere of 5% CO_2_ at 37 °C. Subsequently, the same volume of MTT extraction solution (10% SDS and 0.01 N HCl) was added to the culture medium, and the absorbance at 560 nm was measured after 16 h at room temperature.

#### 2.4.3. Enzyme-Linked Immunosorbent Assay (ELISA)

The level of brain-derived neurotrophic factor (BDNF) was measured using the Mature BDNF ELISA Kit (Wako, FUJIFILM Wako Pure Chemical Corporation, Japan). The experimental procedure followed the manual accompanying the kit. In brief, the culture medium of SH-SY5Y cells at 28 days after induction of differentiation was added to the ELISA plate and incubated at 25 °C for 2 h. After washing, subsequently, a biotin-conjugated specific antibody was incubated at 25 °C for 1 h to bind the captured BDNF. Then, peroxidase-conjugated streptavidin solution was added and incubated at 25 °C for 30 min. Unbound conjugates were removed by subsequent washing. Plates were incubated with TMB (3,3′,5,5′-Tetramethylbenzidine), and the absorbance at 450 nm was measured. All samples were assayed in triplicate.

### 2.5. Data Analysis

Statistical analyses for the behavioral, IHC, MTT assay, and ELISA experiments were performed using SPSS Statistics (version 21.0; IBM). Data of behavioral, MTT assay, and ELISA experiments were assessed by analysis of variance (ANOVA), while those of IHC were analyzed by two-way ANOVA (SPSS Statistics, version 21.0; IBM). The results are presented as the average ± standard deviation (SD). Post hoc tests comprising individual comparisons were performed using the Bonferroni test. Spearman’s test was conducted to assess the correlation between the number of nociceptive neural responses in the brain versus pain-like behaviors and was separately conducted using the results obtained from Rice-*koji*- and EGT-treated groups. Time spent on formalin-evoked licking behaviors for 45 min observation was compared with formalin-evoked c-Fos and FosB expressions in the PVN, NRM, and DH. In the case of the DH, the numbers of c-Fos- and FosB-positive cells in laminae I–II and III–V were combined. The sample size in each treatment group is shown in the figure legend. A statistical level of *p* < 0.05 was considered significant.

## 3. Results

### 3.1. Measurement of Ergothioneine (EGT)

We analyzed our Rice-*koji* extract (Rice-*koji*) by LC-MS/MS to measure how much EGT was contained. The result revealed that 1.0 g of Rice-*koji* contained 13.0 μg of EGT. The dose of EGT contained in Rice-*koji* (High dose), which was administered to each mouse, was equivalent to that of 0.1 mg/kg EGT. The data of the chromatogram are presented in Supplementary Materials Figure S1.

### 3.2. Effects of Rice-koji and EGT on Anxiety and Recognition

#### 3.2.1. Immobility Time during FST

**Figure 2:**
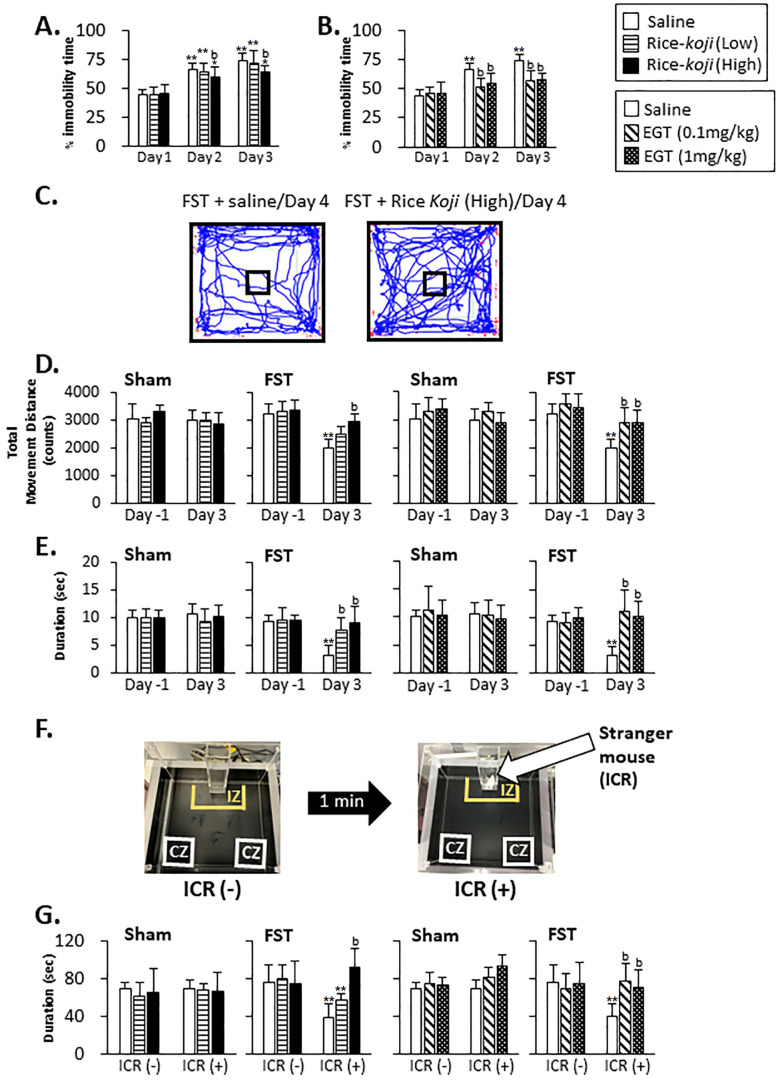
Effect of daily administration of Rice-*koji* and EGT on anxiety-like behaviors induced by repeated forced swim stress treatment (FST). Data summary for the effect of Rice-*koji* (**A**) and EGT (**B**) on time spent on non-swimming behaviors (immobility time). Examples of the open field (OF) area with the mouse trail are depicted (**C**), and the small square located in the middle of the OF area was arbitrarily designated as the center area. The data summary for the effect of Rice-*koji* and EGT on the total movement distance (**D**) and the time spent in the center area (**E**) during the OF test. The experimental procedures for the SI test (**F**). The SI test consisted of two sessions in the absence (ICR (−), 2.5 min) and presence (ICR (+), 2.5 min) of a stranger mouse placed inside the perforated acrylic enclosure in the middle of the interaction zone (IZ), and the time spent within the IZ was recorded. Data summary for the effect of Rice-*koji* and EGT on time spent within the IZ for Sham and FST mice (**G**). * *p* < 0.05, ** *p* < 0.01 versus Day 1 (**A**,**B**), Day −1 (**D**,**E**), or ICR (−) (**G**) with the same treatment group. ^b^ *p* < 0.01 versus the saline-treated group after FST (**A**,**D**,**E**) or that with ICR (+) (**G**). The sample size was analyzed in the FST: FST + saline = 20; FST + Rice-*koji* (Low dose) = 20; FST + Rice-*koji* (High dose) = 20; FST + EGT (0.1 mg/kg) = 20; FST + EGT (1.0 mg/kg) = 20. The sample size was analyzed in the OF and SI test: Sham + saline = 10; Sham + Rice-*koji* (Low dose) = 7; Sham + Rice-*koji* (High dose) = 8; Sham + EGT (0.1 mg/kg) = 10; Sham + EGT (1.0 mg/kg) = 10; FST + saline = 10; FST + Rice-*koji* (Low dose) = 10; FST + Rice-*koji* (High dose) = 10; FST + EGT (0.1 mg/kg) = 9; FST + EGT (1.0 mg/kg) = 10.

The percentage of immobility time during 5 min observation on Day 2 and Day 3 in the saline-treated group was significantly increased compared with that on Day 1 (Figure 2A,B). The percentage of immobility time measured in mice treated with Rice-*koji* (High dose, *p* < 0.01) and EGT (0.1 and 1.0 mg/kg, *p* < 0.01) on Day 2 and Day 3 was significantly smaller than that in those treated with saline (Figure 2A,B).

#### 3.2.2. OF Test

Figure 2C displays examples of travel trajectories of mice in the OF. In Sham mice, total movement distance in the field (Figure 2D) and time spent in the center area (Figure 2E) measured on Day −1 were similar to those on Day 3 regardless of saline, Rice-*koji*, and EGT treatments.

In FST mice treated with saline, total movement distance (*p* < 0.01) and time spent in the center area (*p* < 0.01) on Day 3 were significantly decreased compared with on Day −1. In Rice-*koji* (High dose)- and EGT (0.1 and 1.0 mg/kg)-treated groups, total movement distance (*p* < 0.01) and time spent in the center area (*p* < 0.01) were significantly greater than in the saline-treated group.

#### 3.2.3. SI Test

On Day 4, time spent in the interaction zone (IZ) by Sham mice was similar between treatment groups in the absence (−) and presence (+) of the ICR mouse (*p* > 0.1, Figure 2F,G). In FST mice treated with saline and Rice-*koji* (Low dose), time spent in the IZ in the presence (+) of the ICR mouse was significantly shorter than in the absence (−) of the ICR mouse (*p* < 0.01, Figure 2G); however, in the presence of ICR mouse, time spent in the IZ by FST mice treated with Rice-*koji* (High dose) and EGT (0.1 and 1.0 mg/kg) was significantly longer than that spent by FST mice with saline (*p* < 0.01, Figure 2G).

#### 3.2.4. EPM Test

**Figure 3:**
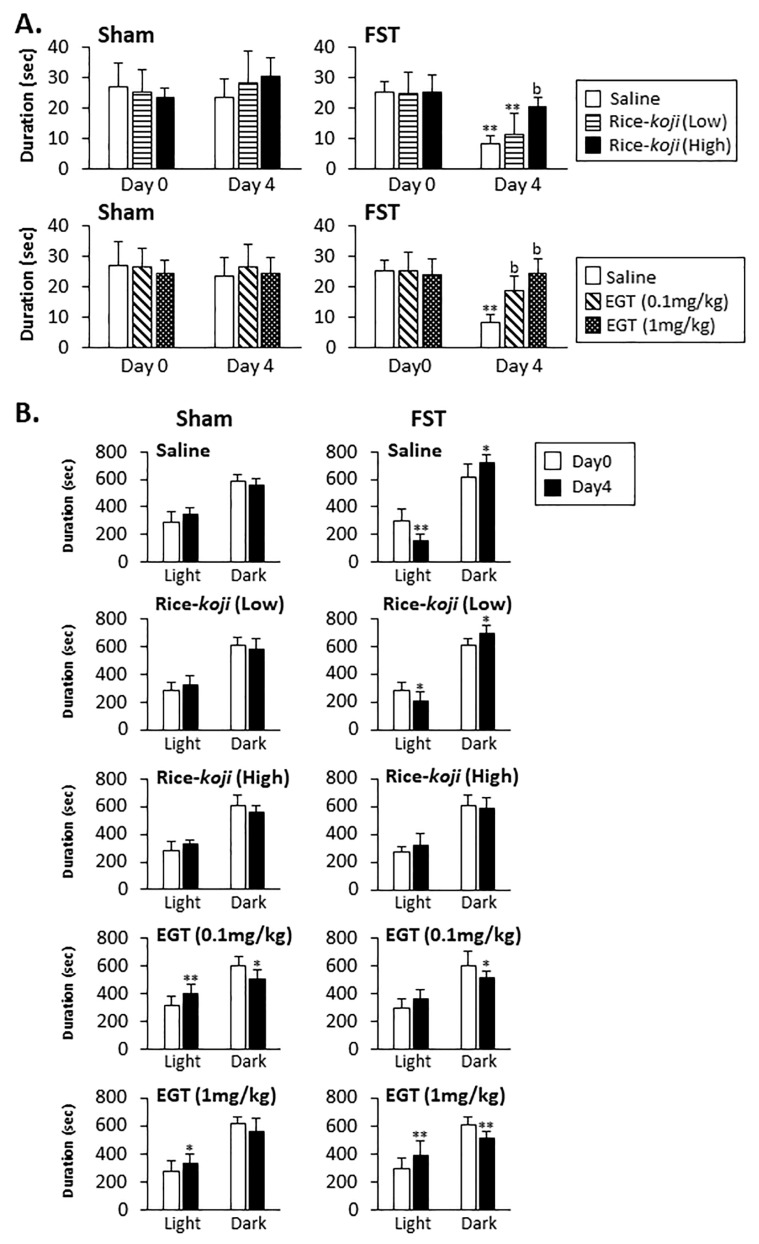
Summary data for the effect of Rice-*koji* and EGT on anxiety-like behaviors. The elevated plus maze and dark and light box tests were conducted in Sham and FST mice on Day 0 and Day 4. Time spent within the open arms (**A**) and the light and dark room (**B**) was compared between Day 0 and Day 4. * *p* < 0.05, ** *p* < 0.01 versus Day 0 in the same treatment group. ^b^ *p* < 0.01 versus the saline-treated group after FST. Sample size: Sham + saline = 10; Sham + Rice-*koji* (Low dose) = 11; Sham + Rice-*koji* (High dose) = 10; Sham + EGT (0.1 mg/kg) = 10; Sham + EGT (1 mg/kg) = 10; FST + saline = 10; FST + Rice-*koji* (Low dose) = 10; FST + Rice-*koji* (High dose) = 10; FST + EGT (0.1 mg/kg) = 10; FST + EGT (1.0 mg/kg) = 10.

In Sham mice, time spent in the open arms on Day 0 and Day 4 was similar between groups regardless of treatments of saline, Rice-*koji*, and EGT (Figure 3A). In FST mice, time spent in the open arms by mice in the saline (*p* < 0.01) and Rice-*koji* (Low dose, *p* < 0.01) groups on Day 4 was significantly decreased compared with on Day 0 (Figure 3A). On Day 4, the time spent in the open arms by FST mice treated with Rice-*koji* (High doses) and EGT (0.1 and 1.0 mg/kg) was significantly longer than that spent by the saline group (*p* < 0.01, Figure 3A).

#### 3.2.5. DL Test

In Sham mice treated with saline and Rice-*koji*, time spent in the light chamber on Day 4 was similar to that on Day 0 (*p* > 0.1), while EGT (0.1 mg/kg, *p* < 0.01 and 1.0 mg/kg, *p* < 0.05, Figure 3B, left panels) significantly increased the time spent in the light chamber on Day 4 compared with on Day 0 (*p* < 0.01).

In FST mice treated with saline (*p* < 0.01) and Rice-*koji* (Low dose, *p* < 0.05) (Figure 3B, right panel), time spent in the light chamber on Day 4 significantly decreased compared with that on Day 0. On the other hand, time spent in the light chamber by FST mice treated with Rice-*koji* (High dose) and EGT (0.1 mg/kg) on Day 4 was similar to that on Day 0 (*p* > 0.1, Figure 3B). In FST mice treated with EGT (1.0 mg/kg), time spent in the light chamber on Day 4 was significantly increased compared with that on Day 0 (*p* < 0.01, Figure 3B).

#### 3.2.6. OR Test

**Figure 4:**
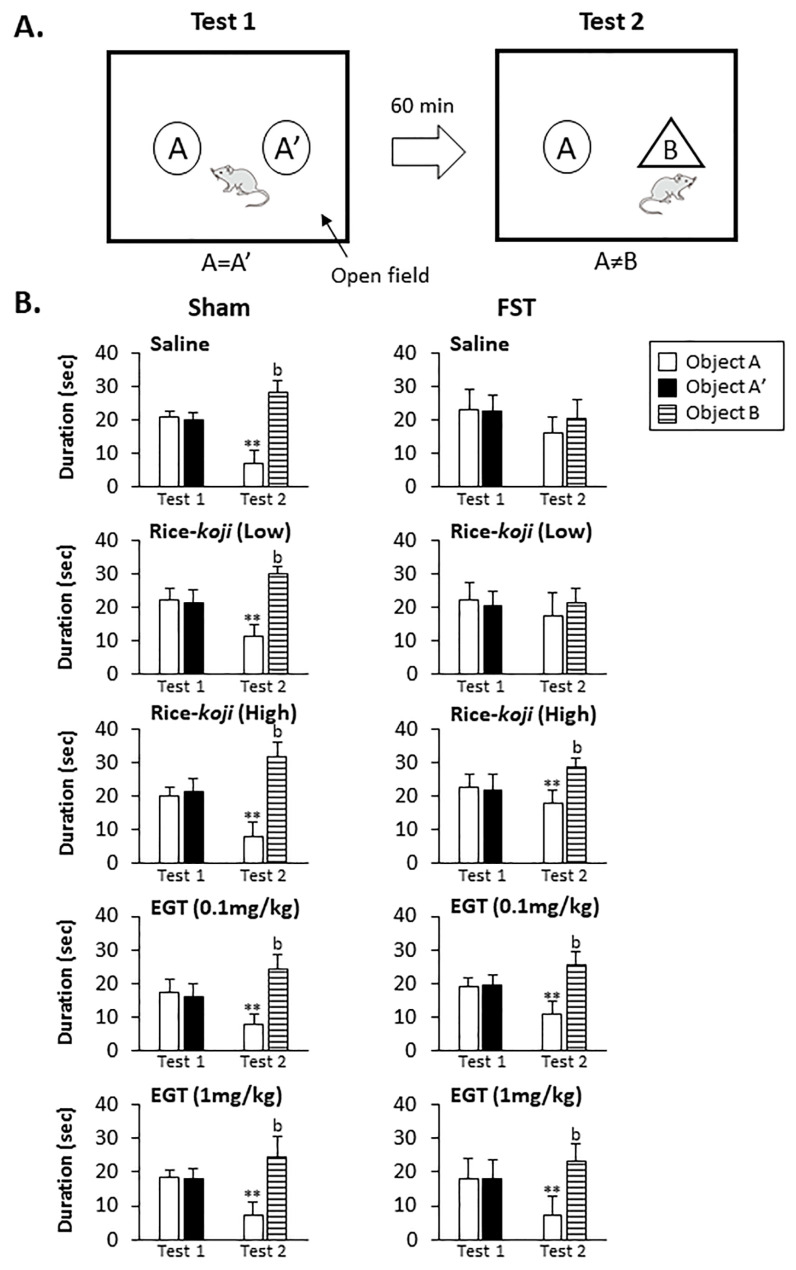
Experimental procedures (**A**) and summary data (**B**) for the effect of Rice-*koji* and EGT on the ability of object recognition (OR) on Day 4. The OR test consisted of two sessions, Tests 1 and 2 (**A**). First, in Test 1, mice were placed in the open field and allowed to discover two similar objects named A and A’. A and A’ are the same object, and time spent in the A and A’ areas was measured for 5 min before the mice were returned to their cage. Sixty minutes after Test 1, Test 2 was conducted for 5 min, and object A’ was replaced with novel object B. Mice were brought to the testing area again and exposed to a familiar (object A) and novel object (object B). The time spent in the A and B areas was recorded and then compared between each area (**B**). ** *p* < 0.01 versus time spent in the A area in Test 1. ^b^ *p* < 0.01 versus time spent in the A area in Test 2. Sample size: Sham + saline = 10; Sham + Rice-*koji* (Low dose) = 6; Sham + Rice-*koji* (High dose) = 10; Sham + EGT (0.1 mg/kg) = 10; Sham + EGT (1.0 mg/kg) = 10; FST + saline = 10; FST + Rice-*koji* (Low dose) = 10; FST + Rice-*koji* (High dose) = 8; FST + EGT (0.1mg/kg) = 9; FST + EGT (1.0 mg/kg) = 10.

Figure 4A shows an illustration showing the structure of the OR test. Figure 4B displays the data for time spent in each area on Day 4.

In Test 1, time spent in “the A area” was similar to that spent in “the A′ area” for both Sham (*p* > 0.1) and FST (*p* > 0.1) mice. In Test 2, the time spent on the novel object (in “the B area”) by Sham mice treated with saline, Rice-*koji*, and EGT was significantly greater than time spent in “the A area” (*p* < 0.01, Figure 4B, left panels). In FST mice treated with saline and Rice-*koji* (Low dose), time spent on the novel object (in “the B area”) was similar to that spent in “the A area” (Test 2, *p* > 0.1, Figure 4B, right panels). However, the time spent in “the B area” by FST mice treated with Rice-*koji* (High dose, *p* < 0.01) and EGT (0.1 and 1.0 mg/kg, *p* < 0.01) was significantly greater than that spent in “the A area” (Figure 4B, right panels).

### 3.3. Effects of Rice-koji and EGT on Pain-like Behaviors

#### 3.3.1. Heat Stimulation

**Figure 5:**
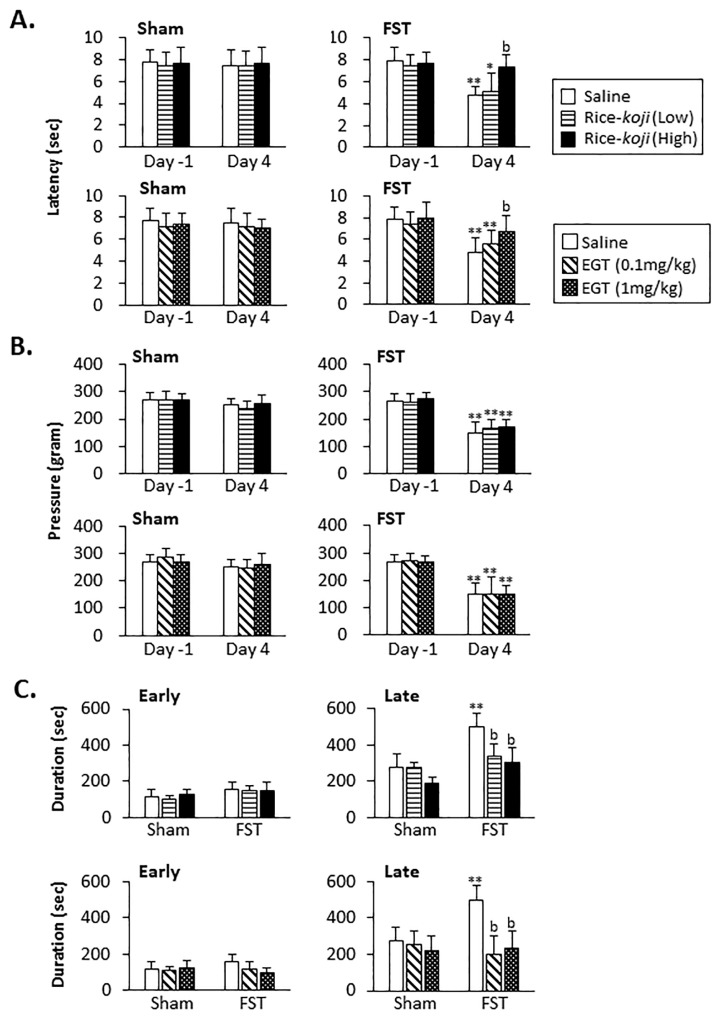
Summary data for the effect of Rice-*koji* and EGT on the hindpaw nociception evoked by mechanical pressure (**A**), thermal (**B**), and formalin (**C**) stimulation in Sham and FST mice. The latency of thermal stimulation and the threshold of withdrawal reflex of the left hindpaw were measured on Days −1 and 4. Time spent on licking behaviors of the affected hindpaw was measured in the early (0–9 min) and late (9–45 min) phases. The formalin test was conducted in Experiment 1 (*n* = 79), while thermal and mechanical tests were conducted in Experiment 2 (*n* = 96). * *p* < 0.05, ** *p* < 0.01 versus Day −1 (**A**,**B**) or Sham (**C**) in the same treatment group. ^b^ *p* < 0.01 versus the saline-treated group after FST. Sample size: Sham + saline = 19 (9); Sham + Rice-*koji* (Low dose) = 19 (7); Sham + Rice-*koji* (High dose) = 20 (7); Sham + EGT (0.1 mg/kg) = 17 (7); Sham + EGT (1.0 mg/kg) = 17 (7); FST + saline = 21 (10); FST + Rice-*koji* (Low dose) = 18 (8); FST + Rice-*koji* (High dose) = 18 (8); FST + EGT (0.1 mg/kg) = 18 (8); FST + EGT (1.0 mg/kg) = 18 (8). ( ): the number of mice employed in Experiment 1.

In Sham mice, the withdrawal latency was similar between Day −1 and Day 4 regardless of Rice-*koji* and EGT treatments (*p* > 0.1, Figure 5A). In FST mice treated with saline (*p* < 0.01), Rice-*koji* (Low dose, *p* < 0.05), and EGT (0.1 mg/kg, *p* < 0.01), the withdrawal latency on Day 4 was significantly decreased compared with that on Day −1. However, on Day 4, the withdrawal latency in FST mice treated with Rice-*koji* (High dose) and EGT (1.0 mg/kg) was significantly longer than in those treated with saline (*p* < 0.01, Figure 5A).

#### 3.3.2. Mechanical Stimulation

In Sham mice, the withdrawal threshold was similar between Day −1 and Day 4 regardless of treatments (*p* > 0.1, Figure 5B). In FST mice treated with saline (*p* < 0.01), Rice-*koji* (*p* < 0.01), and EGT (*p* < 0.01), the withdrawal threshold on Day 4 was significantly decreased compared with that on Day 0, with no significant difference between treatment groups (*p* > 0.1, Figure 5B).

#### 3.3.3. Formalin Test

In the early phase, the time spent on licking behaviors was similar between Sham and FST mice (*p* > 0.1). Rice-*koji* and EGT did not change such nocifensive behaviors in Sham and FST mice (*p* > 0.1, Figure 5C, left panels).

In the late phase, time spent on licking behaviors by saline-treated FST mice was significantly longer than that spent by Sham mice (*p* < 0.01, Figure 5C), while licking behavioral time in FST mice treated with Rice-*koji* (Low and High doses) and EGT (0.1 and 1.0 mg/kg) was significantly shorter than in those treated with saline. (*p* < 0.01, Figure 5C, right panels). In Sham mice, neither treatment changed the nocifensive behaviors (*p* > 0.1).

### 3.4. c-Fos and FosB Immunohistochemistry

#### 3.4.1. PVN

**Figure 6:**
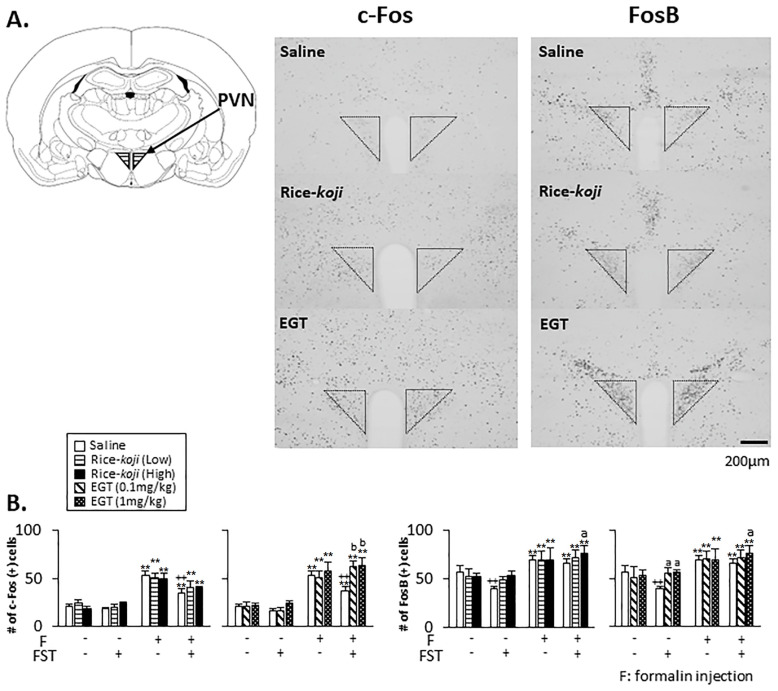
Effect of Rice-*koji* and EGT on neural activities indicated by c-Fos and FosB immunoreactivities in the paraventricular nucleus of the hypothalamus associated with psychological stress and nociceptive responses in the absence (formalin (−)) or presence (formalin (+)) of formalin injection into the hindpaw. (**A**) Example of microphotographs for c-Fos and FosB immunoreactivities in FST mice with formalin injection with or without Rice-*koji* or EGT. (**B**) Histograms display the average number of c-Fos- and FosB-positive cells/section. ** *p* < 0.01 versus formalin (−) group in the same treatment group with or without FST. ^a^ *p* < 0.05, ^b^ *p* < 0.01 versus saline-treated FST group with or without formalin injection. ++ *p* < 0.01 versus saline-treated Sham group with or without formalin injection. Sample size: Sham + saline + formalin (−) = 6; Sham + Rice-*koji* (Low dose) + formalin (−) = 4; Sham + Rice-*koji* (High dose) + formalin (−) = 6; Sham + saline + formalin (+) = 8; Sham + Rice-*koji* (Low dose) + formalin (+) = 6; Sham +Rice-*koji* (High dose) + formalin (+) = 6; FST + saline + formalin (−) = 5; FST + Rice-*koji* (Low dose) + formalin (−) = 4; FST + Rice-*koji* (High dose) + formalin (−) = 6; FST + saline + formalin (+) = 9; FST + Rice-*koji* (Low dose) + formalin (+) = 6; FST + Rice-*koji* (High dose) + formalin (+) = 6; Sham + EGT (0.1 mg/kg) + formalin (−) = 5; Sham + EGT (1.0 mg/kg) + formalin (−) = 6; Sham + EGT (0.1 mg/kg) + formalin (+) = 5; Sham + EGT (1.0 mg/kg) + formalin (+) = 8; FST + Rice-*koji* (Low dose) + formalin (−) = 5; FST + Rice-*koji* (High dose) + formalin (−) = 6; FST + EGT (0.1 mg/kg) + formalin (+) = 6; FST + ERFO (1.0 mg/kg) + formalin (+) = 6.

In the absence of formalin injection (Figure 6B, left panel), the number of c-Fos-positive cells in saline-treated FST mice was similar to that in Sham mice (*p* > 0.1), and Rice-*koji* (*p* > 0.1) and EGT (*p* > 0.1) did not change the number of c-Fos-positive cells in Sham and FST mice. In saline-treated groups (Figure 6B, right panel), the number of FosB-positive cells in FST mice was significantly smaller than in Sham mice (*p* < 0.01). Further, in the FST group, the number of FosB-positive cells was significantly greater in EGT (0.1 and 1.0 mg/kg)-treated mice than in saline-treated mice (*p* < 0.05).

In the presence of formalin injection (Figure 6A), the number of c-Fos-positive (Figure 6B, left panel) and FosB-positive (Figure 6B, right panel) cells in saline- (*p* < 0.01), Rice-*koji*- (*p* < 0.01), and EGT-treated (*p* < 0.01) groups was greater than in the same treatment groups without formalin injection. In the saline-treated group, the number of c-Fos-positive cells in FST mice was significantly smaller than that in Sham mice (*p* < 0.01), whereas the number of c-Fos-positive cells in EGT (0.1 mg/kg, *p* < 0.01; 1.0 mg/kg, *p* < 0.01)-treated mice was significantly greater than in saline-treated FST mice. The number of FosB-positive cells in FST mice treated with Rice-*koji* (High dose, *p* < 0.05) and EGT (1.0 mg/kg, *p* < 0.05) was significantly greater than in saline-treated FST mice.

#### 3.4.2. NRM

**Figure 7:**
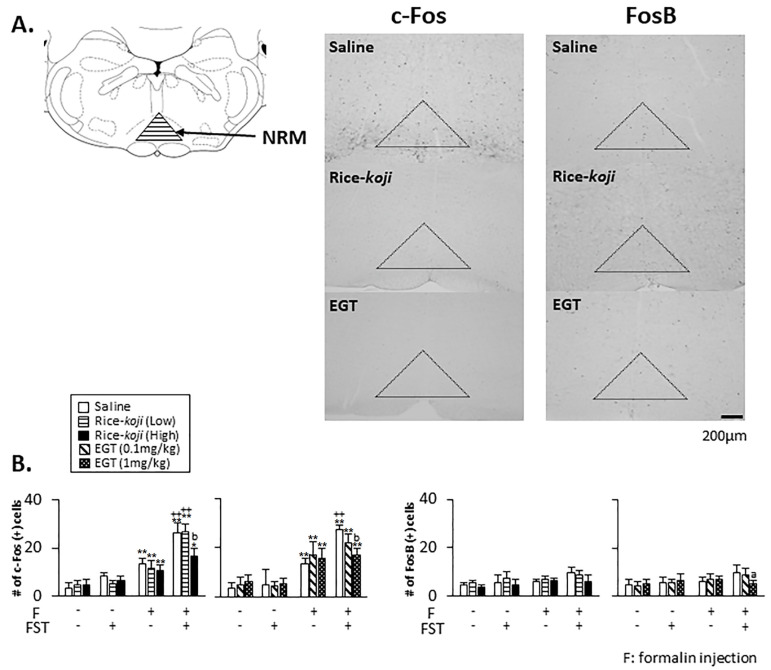
Effect of Rice-*koji* and EGT on neural activities indicated by c-Fos and FosB immunoreactivities in the nucleus raphe magnus in the caudal brainstem associated with psychological stress and nociceptive responses in the absence (formalin (−)) or presence (formalin (+)) of formalin injection into the hindpaw. (**A**) Example of microphotographs for c-Fos and FosB immunoreactivities in FST mice with formalin injection with or without Rice-koji or EGT. (**B**) Histograms displaying the average number of c-Fos- and FosB-positive cells/section. Animals were selected at random. * *p* < 0.05, ** *p* < 0.01 versus formalin (−) group in the same treatment group with or without FST. ^a^ *p* < 0.05, ^b^ *p* < 0.01 versus saline-treated FST group with formalin injection. ++ *p* < 0.01 versus saline-treated Sham group with formalin injection. Sample size: Sham + saline + formalin (−) = 6; Sham + Rice-*koji* (Low dose) + formalin (−) = 4; Sham + Rice-*koji* (High dose) + formalin (−) = 6; Sham + saline + formalin (+) = 8; Sham + Rice-*koji* (Low dose) + formalin (+) = 6; Sham +Rice-*koji* (High dose) + formalin (+) = 6; FST + saline + formalin (−) = 5; FST + Rice-*koji* (Low dose) + formalin (−) = 4; FST + Rice-*koji* (High dose) + formalin (−) = 6; FST + saline + formalin (+) = 9; FST + Rice-*koji* (Low dose) + formalin (+) = 6; FST + Rice-*koji* (High dose) + formalin (+) = 6; Sham + EGT (0.1 mg/kg) + formalin (−) = 5; Sham + EGT (1.0 mg/kg) + formalin (−) = 6; Sham + EGT (0.1 mg/kg) + formalin (+) = 5; Sham +EGT (1.0 mg/kg) + formalin (+) = 8; FST + Rice-*koji* (Low dose) + formalin (−) = 5; FST + Rice-*koji* (High dose) + formalin (−) = 6; FST + EGT (0.1 mg/kg) + formalin (+) = 6; FST +ERFO (1.0 mg/kg) + formalin (+) = 6.

In the absence of formalin injection (Figure 7B), the number of c-Fos- (*p* > 0.1) and FosB-positive (*p* > 0.1) cells in saline-treated FST mice was similar to that in Sham mice. Rice-*koji* (*p* > 0.1) and EGT (*p* > 0.1) did not alter the number of c-Fos and FosB expressions in Sham and FST mice.

In the presence of formalin injection (Figure 7A), the number of c-Fos-positive cells in saline, Rice-*koji*, and EGT was significantly greater than in the same treatment groups without formalin injection in Sham and FST mice (Figure 7B, left panel). Further, the number of c-Fos-positive cells in saline-treated FST mice was significantly greater than in saline-treated Sham mice (*p* < 0.01). The number of c-Fos-positive cells in FST mice treated with Rice-*koji* (High dose) and EGT (1.0 mg/kg) was significantly smaller than in the saline group (*p* < 0.01, Figure 7B, left panel). In Sham mice, neither treatment affected c-Fos expressions in the NRM (*p* > 0.1, Figure 7B, left panel). The FosB expressions in Sham and FST mice were not changed by formalin injection (*p* > 0.1); however, the number of FosB-positive cells in EGT (1.0 mg/kg)-treated FST mice was significantly smaller than in the saline group (*p* < 0.05, Figure 7B, right panel).

#### 3.4.3. DH

**Figure 8:**
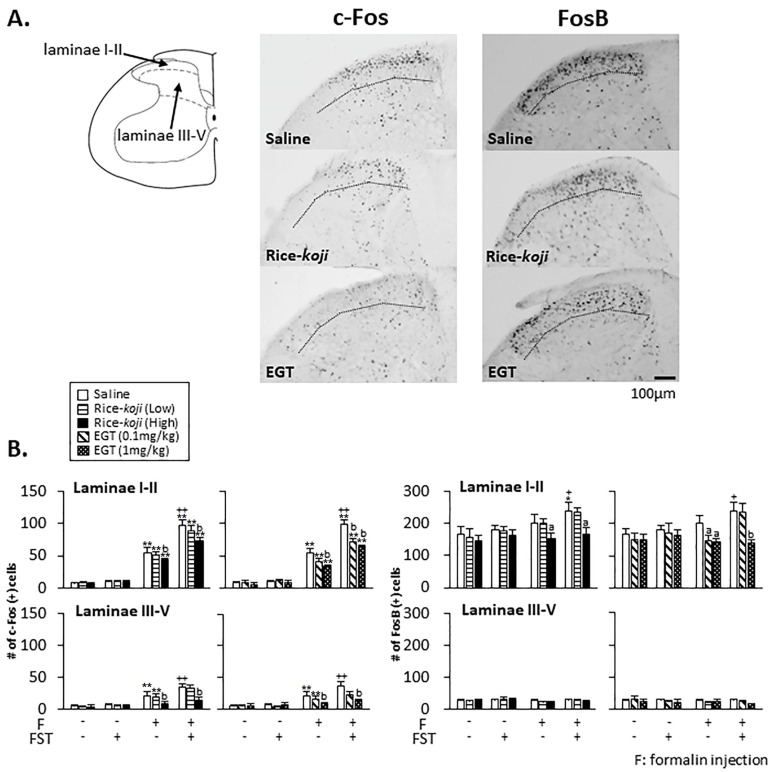
Effect of Rice-*koji* and EGT on neural activities indicated by c-Fos and FosB immunoreactivities in the laminae I–II and III–V at the dorsal horn of the lumbar spinal cord associated with psychological stress and nociceptive responses in the absence (formalin (−)) or presence (formalin (+)) of formalin injection into the hindpaw. (**A**) Example of microphotographs for c-Fos and FosB immunoreactivities in FST mice with formalin injection with or without Rice-*koji* or EGT. (**B**) Histograms displaying the average number of c-Fos- and FosB-positive cells/section. Cell counts were assessed on the ipsilateral (left) side. Animals were selected at random. * *p* < 0.05, ** *p* < 0.01 versus formalin (−) group in the same treatment group with or without FST. ^a^ *p* < 0.05, ^b^ *p* < 0.01 versus saline-treated group with or without FST. + *p* < 0.05, ++ *p* < 0.01 versus saline-treated Sham group with formalin injection. Sample size: Sham + saline + formalin (−) = 6; Sham + Rice-*koji* (Low dose) + formalin (−) = 4; Sham + Rice-*koji* (High dose) + formalin (−) = 6; Sham + saline + formalin (+) = 8; Sham + Rice-*koji* (Low dose) + formalin (+) = 6; Sham +Rice-*koji* (High dose) + formalin (+) = 6; FST + saline + formalin (−) = 5; FST + Rice-*koji* (Low dose) + formalin (−) = 4; FST + Rice-*koji* (High dose) + formalin (−) = 6; FST + saline + formalin (+) = 9; FST + Rice-*koji* (Low dose) + formalin (+) = 6; FST + Rice-*koji* (High dose) + formalin (+) = 6; Sham + EGT (0.1 mg/kg) + formalin (−) = 5; Sham + EGT (1.0 mg/kg) + formalin (−) = 6; Sham + EGT (0.1 mg/kg) + formalin (+) = 5; Sham + EGT (1.0 mg/kg) + formalin (+) = 8; FST + Rice-*koji* (Low dose) + formalin (−) = 5; FST + Rice-*koji* (High dose) + formalin (−) = 6; FST + EGT (0.1 mg/kg) + formalin (+) = 6; FST + ERFO (1.0 mg/kg) + formalin (+) = 6.

In the absence of formalin injection, FST alone did not change c-Fos (Figure 8B, left panel) and FosB (Figure 8B, right panel) expressions in the DH compared with in Sham mice. Rice-*koji* and EGT did not change c-Fos and FosB expressions in Sham and FST mice (*p* > 0.1).

In the presence of formalin injection (Figure 8A), the number of c-Fos-positive cells in laminae I–II (*p* < 0.01) and III–V (*p* < 0.01) in saline-treated FST mice was significantly greater than in Sham mice (Figure 8B, left panel). The number of c-Fos-positive cells in both laminae in Sham (*p* < 0.01) and FST (*p* < 0.01) mice treated with Rice-*koji* (High dose) and EGT (1.0 mg/kg) was significantly smaller than that in those treated with saline (Figure 8B, left panel).

The number of formalin-evoked FosB-positive cells in laminae I–II in saline-treated FST mice was greater than in Sham mice (*p* < 0.05, Figure 8B, right panel). In both Sham and FST mice, the number of FosB expressions in Rice-*koji* (High dose: *p* < 0.05 for Sham, *p* < 0.05 for FST)- and EGT (1.0 mg/kg: *p* < 0.05 for Sham, *p* < 0.01 for FST)-treated groups was smaller than in the saline-treated group in laminae I–II. However, in laminae III–V, FosB expressions were not changed by Rice-*koji* and EGT (*p* > 0.1).

**Figure 9:**
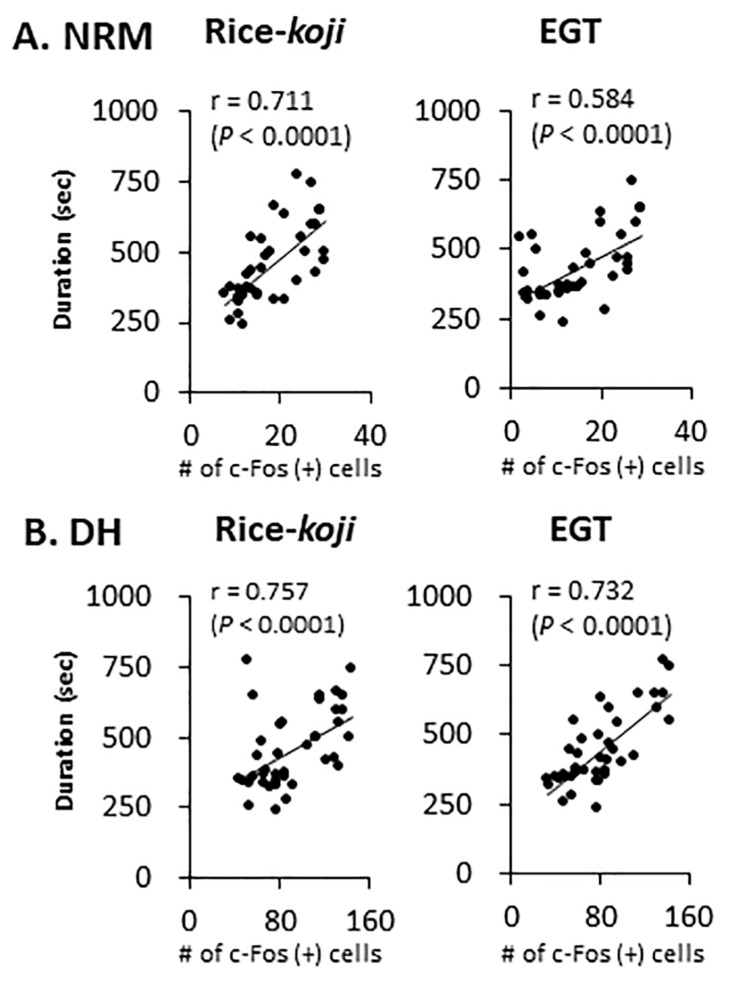
Scatter plots displaying the correlation between the number of c-Fos-positive cells in the nucleus raphe magnus (NRM) (**A**) and lumbar spinal dorsal horn (DH) (**B**) and time spent in formalin-evoked nocifensive behaviors. Sample size: all mice received a hindpaw injection of formalin. Sham + saline = 8; Sham + Rice-*koji* (Low dose) = 6; Sham + Rice-*koji* (High dose) = 6; FST + saline = 9; FST + Rice-*koji* (Low dose) = 6; FST + Rice-*koji* (High dose) = 6; Sham + EGT (0.1 mg/kg) = 6; Sham + EGT (1.0 mg/kg) = 6; FST + EGT (0.1 mg/kg) = 6; FST + EGT (1.0 mg/kg) = 6. Abbreviations: r = Spearman’s rank correlation coefficient.

Spearman’s test was conducted to evaluate the possible correlation between neural responses in the brain and pain-like behaviors. Figure 9 demonstrates the relationship of c-Fos expressions with pain-like behavioral times related to formalin stimulation.

The c-Fos expression in the DH (r = 0.76, *p* < 0.0001) and NRM (r = 0.71, *p* < 0.0001) was significantly correlated with pain-like behaviors in the Rice-*koji* and EGT treatment groups (Figure 9A,B). The FosB expression was significantly correlated with pain-like behaviors in the DH (r = 0.63, *p* < 0.0001) but not in the NRM (r = 0.11, *p* > 0.5, not shown in the figures). Further, formalin-evoked c-Fos expressions in the PVN (r = −0.49, *p* < 0.004) were negatively correlated with pain-like behaviors; however, FosB expressions were not (r = −0.25, *p* < 0.17, not shown in the figures).

### 3.5. MTT Assay

**Figure 10:**
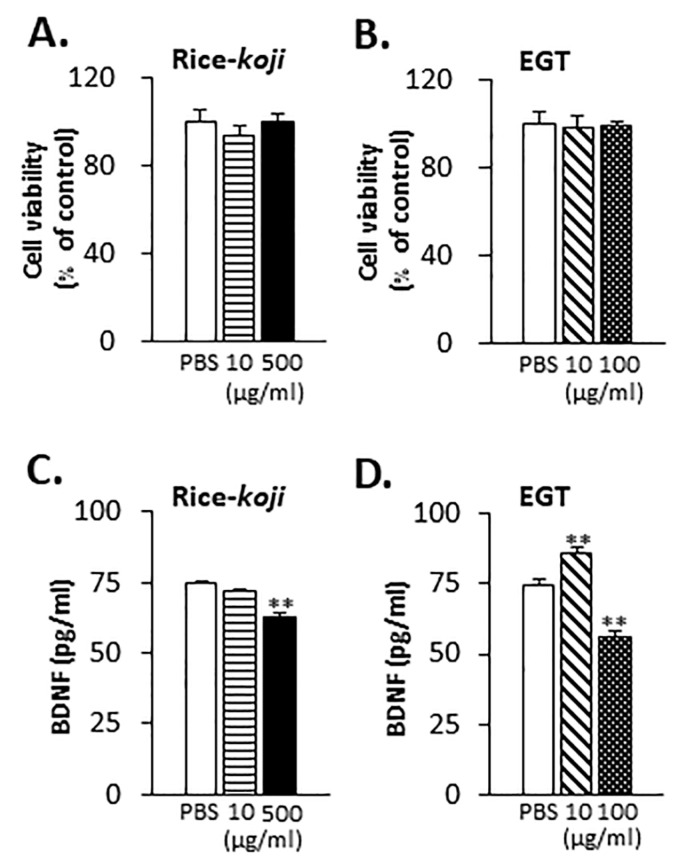
Summary data for the effect of Rice-*koji* and EGT on cell viability (**A**,**B**) measured by the MTT assay and the level of brain-derived neurotrophic factor (pg/mL) measured by ELISA in SH-SY5Y cells (**C**,**D**). Results of each treatment are shown by average values collected from three samples, which were repeated three times. ** *p* < 0.01 versus PBS.

To examine the effect of Rice-*koji* and EGT on the viability of neural cells in vitro, an MTT assay was performed on SH-SY5Y cells. The results showed that neither treatment changed the cell viability of SH-SY5Y cells compared with PBS (*p* > 0.1, Figure 10A,B).

### 3.6. The Level of BDNF Measured by ELISA

The level of BDNF in SH-SY5Y cells treated with Rice-*koji* (500 µg/mL) was smaller than in those treated with PBS on Day 28 (*p* < 0.01). The level of BDNF in SH-SY5Y cells treated with EGT at doses of 10 and 100 µg/mL was greater (*p* < 0.01) and smaller (*p* < 0.01) than in those treated with PBS, respectively (Figure 10C,D).

## 4. Discussion

In Japan, traditional fermented food made from Rice-*koji* has been a well-known healthy and safe food product for more than 1000 years [4]. Several pieces of scientific research support these notions. For example, Rice-*koji* exerted increased water content in human skin [43], anti-aging effects in human dermal fibroblasts [44], and improvement in defecation frequency [2] and decreased blood pressure [45]. Of note, recent reports also demonstrated that rice-fermented foods can prevent adverse effects induced by psychological stress. Among them, sake and sake lees decreased anxiety- and pain-like responses in psychophysical stress models [8,9]. Similarly, *Asparagus-racemosus*-starter-based rice-fermented foods exerted anti-psychological stress effects in mice [46]. Although these reports demonstrated the anti-stress effects of rice-fermented foods, to our best knowledge, our present study has demonstrated for the first time the preventive effect of Rice-*koji* fermented extracts made by *Aspergillus oryzae* on anxiety, impaired recognition, and nociception using a psychophysically stressed model.

Our results also demonstrated the preventive effects of ergothioneine (EGT) on stress-induced anxiety- and pain-like behaviors, consistent with several previous findings. Oral administration of EGT inhibited depression-like behaviors identified by FST and tail suspension tests [14] and could protect against stress-induced sleep disturbances in rats [47].

These results further allow us to elucidate a possible molecular basis for the preventive effects of Rice-*koji* on anxiety- and pain-like behaviors because EGT is one of the ingredients contained in rice-fermented foods and is a naturally occurring food-derived antioxidant that can regulate neural functions [31]. Oxidative stress is a possible mechanism involved in neuropsychiatric disorders and chronic pain [48]. Our LC-MS analysis confirmed the presence of EGT in Rice-*koji*, and the actual dose of EGT contained in 0.1 mg/kg EGT was the equivalent dose of EGT contained in the High dose of Rice-*koji*. It is noteworthy that daily administration of the High dose of Rice-*koji* or EGT at a dosage of 0.1 mg/kg decreased anxiety- and pain-like behaviors in FST mice. These findings suggest that the inhibitory effects of Rice-*koji* on psychological stress might be mediated through the actions of EGT.

### 4.1. Effects of Rice-koji on Anxiety- and Pain-like Behaviors

This study employed multiple behavioral procedures to evaluate anxiety- and pain-like responses in mice subjected to FST. FST is a traditional procedure used to investigate new antidepressant drugs first described by Porsolt et al. [49]. Several modified FST protocols, including those used in our previous reports [9], have been employed as a psychophysical stress model [17,18,50]. The validity of the FST model, particularly measuring the immobility time as depression-like behaviors similar to psychological distress in humans, has been controversial [18,51]. Our psychological assessments using DL, SI, OF, and EPM tests demonstrated that FST mice exhibited increases in anxiogenic-like behaviors. Further, consistent with ample evidence, the FST model increased the duration of immobility swimming behaviors (Figure 2A,B). The discussion on the validity of immobility time as an indicator of anxiety and depression is beyond the scope of this study.

Anxiolytic effects of Rice-*koji* were determined in FST mice, and a high dose of Rice-*koji* prevented anxiety-like behaviors, identified in all behavioral procedures. However, the effect of a low dose of Rice-*koji* seemed to be less, except for in the OF test. These findings indicated that sensitivity to the detection of anxiety-like behaviors was different between the methodologies for the assessments of behavior. However, the functional significance of the different results between behavior tests remains unclear.

EGT exerted anxiolytic effects in FST mice, identified by all behavioral tests. These findings are consistent with previous reports that demonstrated that EGT reduced the immobility time during FST [14] and increased social interaction behaviors in social defeat stress mice [47]. As a whole, in Sham mice, Rice-*koji* and EGT exerted less of an effect on anxiety-like behaviors, except in the results of the DL test. Of note, EGT increased the time spent in the light chamber on Day 4 (Figure 3B). The precise interpretation of this result’s significance is unclear; however, EGT might have roles in regulating psychological responses even under non-stress conditions. 

We also determined whether Rice-*koji* and EGT could improve cognitive impairment in FST mice. The novel object recognition (OR) behavioral test revealed that time spent at the novel object (“the B area”, Figure 4A, see Test 2) was not prolonged in FST mice treated with saline (Figure 4B, Test 2) compared with that time spent at the familiar one (“the A area”, Test 2); however, Rice-*koji* (High dose) and EGT (0.1 and 1.0 mg/kg) increased the time spent at the novel object (“the B area”) compared with that spent at the familiar one (“the A area”). These findings indicated that Rice-*koji* and EGT might protect FST-induced dysfunction of recognition, while these treatments exerted less of an effect in Sham mice. A clinical study supports our findings, in part, stating that the concentration of EGT in blood serum was reduced in older people with cognitive impairment [52].

In accordance with the effects on anxiety-like behaviors, Rice-*koji* and EGT prevented thermal- and formalin- but not mechanical-stimulation-, evoked nocifensive behaviors in FST mice. These results are consistent with our previous report on the inhibitory roles of another rice-fermented food, sake lees, which could prevent formalin but not mechanical nocifensive behaviors in the hindpaw in FST mice [9]. Although it is unclear why neither Rice-*koji* nor sake lees [9] inhibited mechanical hypersensitivity, the antinociceptive roles of these rice-fermented foods could depend on the modalities of the stimulations.

In Sham mice, Rice-*koji* exerted less of an effect on nocifensive behaviors (Figure 5). This was also the case for sake lees, as shown in our previous reports [9]. The interpretations for these results remain unclear, but the intake period of these rice-fermented foods might have been insufficient to exert anti-nociception. Evidence indicates that the regulatory effect of several substances on body functions depends on the treatment period [53,54]. For example, a longer period of treatment with Pimpinella anisum L. [53] and with ferulic acid [54], one of the ingredients contained in Rice-*koji* [55], was required to decrease systolic blood pressure compared to a high dose of them. The precise basis for the preventive roles of Rice-*koji* on enhanced pain-like behaviors in FST mice is unclear; however, improvements in neural functions in the brain might be due to the actions of EGT contained in Rice-*koji*, in part [56], because Rice-*koji* and EGT prevented enhanced formalin-evoked pain-like behaviors in the late, but not the early, phase in FST mice. It is documented that formalin-evoked nocifensive behaviors in the late phase are mediated by neural changes in the brain rather than by the peripheral nervous system [28,57].

### 4.2. Effects of Rice-koji and EGT on c-Fos and FosB Expressions in the PVN, NRM, and DH

Neural changes in the PVN reflect susceptibility to developing a neuropsychiatric disorder [23,24], and preclinical studies have employed c-Fos and FosB immunoreactivities to elucidate brain function, including that of the PVN. These proteins are transcriptional factors and exert their effects by enhancing or repressing the expression of other genes and are involved in the development and maintenance of anxiety and nociception. In general, acute stress increases c-Fos and FosB expressions in the PVN [58,59], whereas the effect of chronic stress on c-Fos and FosB expressions seems to be bidirectional [60,61,62]. Evidence demonstrated that chronic psychological stress alone decreased c-Fos expressions [61], while antipsychotic drug treatment increased c-Fos expressions in the PVN [63]. 

Likewise, in the absence of formalin injection, FosB expression in the PVN in FST mice was lower than Sham mice, while Rice-*koji* and EGT prevented the reduction of FosB expressions in FST mice (Figure 6, right panel). These findings indicated that decreased anxiety-like behaviors caused by Rice-*koji* and EGT might be due to improvements in neural activities in the PVN.

Although FST alone displayed no effect on c-Fos expressions in the PVN, formalin-evoked c-Fos expressions in saline-treated FST mice were significantly smaller than those in Sham mice (Figure 6, left panel). Further, formalin-evoked c-Fos expressions in the PVN were negatively correlated with pain-like behaviors. These findings indicated that changes in nociceptive neural activities in the PVN could reflect pain-like behaviors. Formalin-evoked FosB expressions in the PVN in saline-treated FST mice were similar to those in Sham mice (Figure 6, right panel), even though FST alone reduced FosB expressions levels compared with those in Sham mice. The interpretations for these findings are unclear; however, FosB expressions related to formalin stimulation might have reached the maximum level. 

In FST mice, EGT, but not Rice-*koji*, increased formalin-evoked c-Fos expressions in the PVN compared with saline treatment (Figure 6, left panel), while Rice-*koji* and EGT increased formalin-evoked FosB expressions (Figure 6, right panel). These findings indicated that the preventive roles of EGT on neural responses were greater than those of Rice-*koji*, and the effects of both treatments on FosB expressions might be greater than on c-Fos expressions. Collectively, despite distinct response patterns of c-Fos and FosB expression, Rice-*koji* and EGT could have preventive roles on the induction of neural changes in the PVN under psychophysical stress conditions.

Psychological stress causes dysfunction of the descending pain controls [64]. Neural changes in the NRM and DH, key components of the descending pain controls, induce chronic pain [9,25,26,65]. In the absence of formalin injection, c-Fos and FosB expressions in the NRM and DH of FST mice were not affected (Figure 7 and Figure 8), although other stress models increased these [27,39]. The c-Fos and FosB expressions associated with psychological stress conditions alone might depend on the types of stress models, which were not affected by Rice-*koji* and EGT. 

Next, the modulatory effect of Rice-*koji* and EGT on neural responses in the NRM and DH was determined in the presence of formalin injection. In Sham mice, Rice-*koji* and EGT decreased c-Fos expressions in laminae I–II and III–V and FosB expression in laminae I–II at the DH (Figure 8B). However, neither treatment changed c-Fos and FosB expressions in the PVN (Figure 6) and NRM (Figure 7) under Sham conditions. These findings indicated that the involvement of Rice-*koji* and EGT in regulating neural responses in the DH seemed to be different from that in the NRM and PVN. Despite decreased c-Fos and FosB expressions in the DH after the treatments with Rice-*koji* and EGT, neither of them decreased pain-like behaviors induced by thermal and formalin stimulation (Figure 5). The precise interpretations for the discrepancy remain unclear, yet decreased c-Fos and FosB expressions in the DH might not have been sufficient to inhibit thermal- and formalin-evoked nocifensive behaviors of Sham mice.

In FST mice, Rice-*koji* (High dose) and EGT reduced formalin-evoked c-Fos expressions in the NRM (Figure 7) and in both laminae at the DH (Figure 8). These results are consistent with the findings that antidepressant agents, sake, and sake lees inhibited enhanced nociceptive responses in the NRM and DH under psychological stress conditions [8,9,26,66]. Further, the inhibitory effects of 0.1 mg/kg EGT on c-Fos expressions in laminae I–II, but not in laminae III–V, indicated that sensitivity of neural responses to EGT might be exerted in a laminae-specific manner in the DH. Despite a small but significant inhibitory effect of EGT (1 mg/kg, Figure 7B, right panel) on FosB expressions in the NRM, the modulatory effect of Rice-*koji* and EGT on FosB expressions was smaller than that on c-Fos expressions. Rice-*koji* and EGT decreased FosB expressions in laminae I–II but not in laminae III–V. The positive correlation of both markers in the DH and that of c-Fos expressions in the NRM evoked by formalin injection supported the notions that nociceptive neural activities in the DH and NRM are significantly correlated with pain-like behaviors [25,64].

### 4.3. Effects of Rice-koji and EGT on the Neural Function of SH-SY5Y Cells

Although elucidation on the precise mechanism of how Rice-*koji* and EGT can regulate c-Fos and FosB expressions in mice is beyond the scope of this study, our in vitro results demonstrated that Rice-*koji* and EGT could have some influences on neural function at the cellular level. Indeed, our ELISA analysis revealed that Rice-*koji* decreased while EGT increased and decreased the BDNF level of the SH-SY5Y cells depending on the concentrations. These findings might offer the possible mechanisms by which Rice-*koji* and EGT affect cellular functions, which could regulate neural responses in the brain. However, the linkage of bidirectional changes in the BDNF level in the SH-SY5Y cells to the regulation of anxiety- and pain-like responses remains unclear because either increases or decreases in the level of BDNF associated with psychological stress could be dependent on the areas in the brain. Psychological stress increased the BDNF level in the anterior cingulate cortex and insular cortex [39] and decreased it in the prefrontal cortex [67] and hippocampus [68].

Further, one might consider that decreases in the BDNF level could be due to the inhibitory effect of Rice-*koji* and EGT on cell viability. It is, however, unlikely because our MTT assay displayed no reduction of it. At this point, it is unclear how the current in vitro results could be connected with the results of the in vivo experiment. However, emphasis is placed on the notion that both treatments exerted a significant effect on the functional activity at the cellular level at least [34].

## 5. Conclusions

Daily administration of Rice-*koji* could alleviate anxiety- and pain-like responses, with changes in neural responses in several areas of the brain under psychophysical stress conditions, and such anti-stress effects might be due to the action of EGT which is contained in Rice-*koji*. This is the first report on the beneficial effects of Rice-*koji* on psychological stress conditions despite the evidence of its traditional use.

## Data Availability

Data are presented in the paper. Raw data will be made available on reasonable request.

## Supplementary Materials

The following supporting information can be downloaded at: https://www.mdpi.com/article/10.3390/nu15183989/s1, Figure S1. Representative chromatograms of liquid chromatography–tandem mass spectrometry (LC-MS/MS) were recorded at 230.1 nm.
