# Supplementary material for: Preventive Roles of Rice-koji Extracts and Ergothioneine on Anxiety- and Pain-like Responses under Psychophysical Stress Conditions in Male Mice
Source: Nutrients. 2023 Sep 14;15(18):3989. doi: 10.3390/nu15183989 (PMC10535605; doi:10.3390/nu15183989)
Supplement: Supplementary file 1 [file nutrients-15-03989-s001.zip › nutrients-2602138-supplementary.pdf]

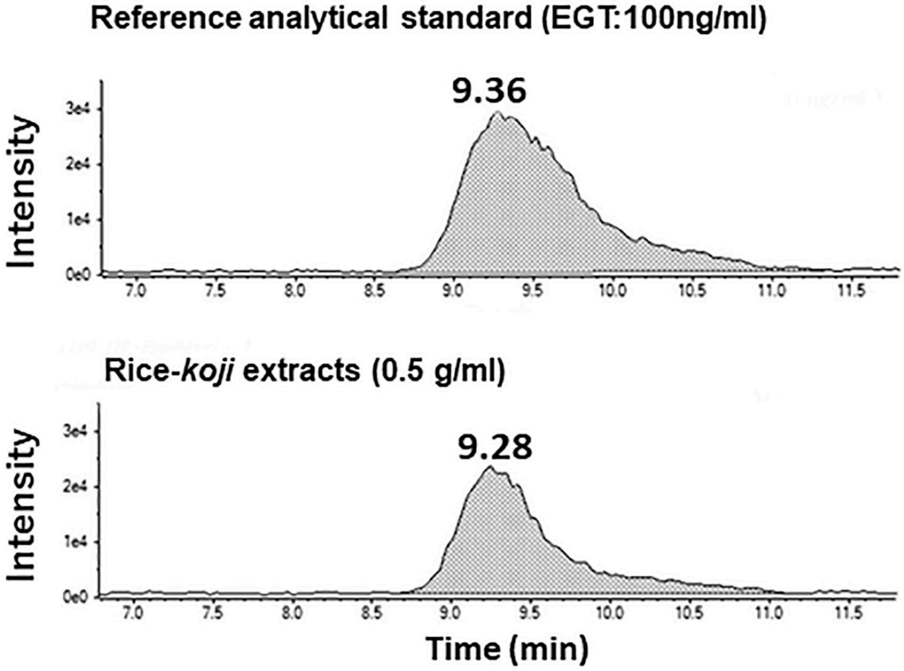

Figure S1. Representative chromatograms of liquid chromatography–tandem mass spectrometry (LC-MS/MS) were recorded at 230.1 nm.
